# Vestigial-like 2 contributes to normal muscle fiber type distribution in mice

**DOI:** 10.1038/s41598-017-07149-0

**Published:** 2017-08-02

**Authors:** Masahiko Honda, Kyoko Hidaka, So-ichiro Fukada, Ryo Sugawa, Manabu Shirai, Masahito Ikawa, Takayuki Morisaki

**Affiliations:** 1 Department of Bioscience and Genetics, National Cerebral and Cardiovascular Center Research Institute, 5-7-1 Fujishirodai, Suita Osaka, 565-8565 Japan; 2 Center for Fundamental Education, The University of Kitakyushu, 4-2-1 Kitagata, Kokura Minami-ku, Kitakyushu, Fukuoka 802-8577 Japan; 3 Department of Immunology, Graduate School of Pharmaceutical Sciences, Osaka University, 1-6 Yamada-oka, Suita Osaka, 565-0871 Japan; 4 Animal Resource Center for Infectious Diseases Research Institute for Microbial Diseases, Osaka University, 3-1 Yamada-oka, Suita, Osaka, 565-0871 Japan; 5 Department of Clinical Engineering, Tokyo University of Technology School of Health Sciences, 5-23-22 Nishi-Kamata, Ota, Tokyo, 144-8535 Japan

## Abstract

Skeletal muscle is composed of heterogeneous populations of myofibers that are classified as slow- and fast-twitch fibers. The muscle fiber-type is regulated in a coordinated fashion by multiple genes, including transcriptional factors and microRNAs (miRNAs). However, players involved in this regulation are not fully elucidated. One of the members of the Vestigial-like factors, Vgll2, is thought to play a pivotal role in TEA domain (TEAD) transcription factor-mediated muscle-specific gene expression because of its restricted expression in skeletal muscles of adult mice. Here, we generated Vgll2 null mice and investigated Vgll2 function in adult skeletal muscles. These mice presented an increased number of fast-twitch type IIb fibers and exhibited a down-regulation of slow type I myosin heavy chain (MyHC) gene, *Myh7*, which resulted in exercise intolerance. In accordance with the decrease in *Myh7*, down-regulation of miR-208b, encoded within *Myh7* gene and up-regulation of targets of miR-208b, Sox6, Sp3, and Purβ, were observed in Vgll2 deficient mice. Moreover, we detected the physical interaction between Vgll2 and TEAD1/4 in neonatal skeletal muscles. These results suggest that Vgll2 may be both directly and indirectly involved in the programing of slow muscle fibers through the formation of the Vgll2-TEAD complex.

## Introduction

Adult mammalian skeletal muscles are made up of heterogeneous populations of myofibers that display distinct contractile and metabolic properties^1–3^. Muscle fiber types are classified as slow- or fast-twitch fibers based on their contractility. Slow-twitch (Type I) fibers are mitochondrial-rich, exhibit oxidative metabolism and fatigue resistance, and express slow isoforms of sarcomeric proteins, including MyHCI, encoded by *Myh7*. In contrast, fast-twitch (Type II) fibers express fast isoforms of sarcomeric proteins, fatigue rapidly, and are subclassified as type IIa, IIx, and IIb based on the expression of *Myh2* (MyHCIIa), *Myh1* (MyHCIIx), and *Myh4* (MyHCIIb), respectively^4^. Type IIa fibers are mitochondrial-rich and exhibit oxidative metabolism. Type IIb fibers have low density of mitochondria and rely on glycolytic metabolism. Type IIx fibers are intermediate. Therefore, the amount and type of MyHC in each skeletal muscle are major indicators of the function of each muscle, including endurance, fatigability, and metabolism.

The process of muscle fiber-type specification is controlled by multiple steps. After embryonic and fetal myogenesis, the pattern of MyHC expression at birth is similar in all skeletal muscles in mice^5, 6^, whereas the pattern of MyHC isoform expression is modified according to physical and functional demands during postnatal life. The muscles then attain a mature phenotype that is functionally distinct. Previous studies identified many transcriptional pathways underlying the regulation of basal-muscle fiber type-specific gene expression, exogenous stimulus-induced fiber type modulation, and myofiber metabolism^7–17^. In addition to protein-encoding genes, miRNAs have emerged as new players in functional modulation of myofibers by participating in orchestrated gene regulation processes^18–21^.

In *Drosophila*, the nuclear protein, Vestigial, is the essential cofactor of Scalloped, a homolog of TEA domain/transcription enhancer factor (TEAD/TEF) family of transcription factors and plays a pivotal role in the development and patterning of the wing^22, 23^. Vestigial is also a necessary cofactor of Scalloped for muscle differentiation^24^. Mammalian vestigial-like 1–4 (Vgll1–4) proteins possess the TEF-1 interaction domain called the tondu (TDU) motif and contribute to the tissue-specific functions of the TEAD factors^25–27^. Of these, Vgll2 (also known as VITO-1) is expressed in the somitic myotome and pharyngeal pouch during mouse development, while it is selectively expressed in skeletal muscle in the adult^25, 26^. Previous studies showed that Vgll2 overexpression augments MyoD-mediated myogenic conversion of 10T1/2 cells^26, 28^. Conversely, knockdown studies using antisense morpholino showed that inhibition of Vgll2 expression attenuates MyHC expression in C2C12 myoblasts and avian limb muscles^29^. In addition, skeletal muscle specific overexpression of TEAD1 induces fast-to-slow fiber-type transition in adult mice^30^. Thus, previous studies have implicated a role for Vgll2 in *in vitro* muscle differentiation, but its *in vivo* function is poorly understood.

In this study, we found that Vgll2-deficient mice exhibited a faster muscle contractile phenotype under basal conditions and significant expression changes of *Myh7/miR-208b* and its downstream targets of transcriptional repressor proteins for slow-twitch fiber in neonatal skeletal muscles. We further provide evidences that Vgll2 forms a protein complex with TEAD1/4. Our study reveals that Vgll2 has potent activity in normal skeletal muscle fiber distribution in both direct and indirect contribution *in vivo*.

## Results

### Preferential Expression of Vgll2 in Slow Muscle

**Figure 1:**
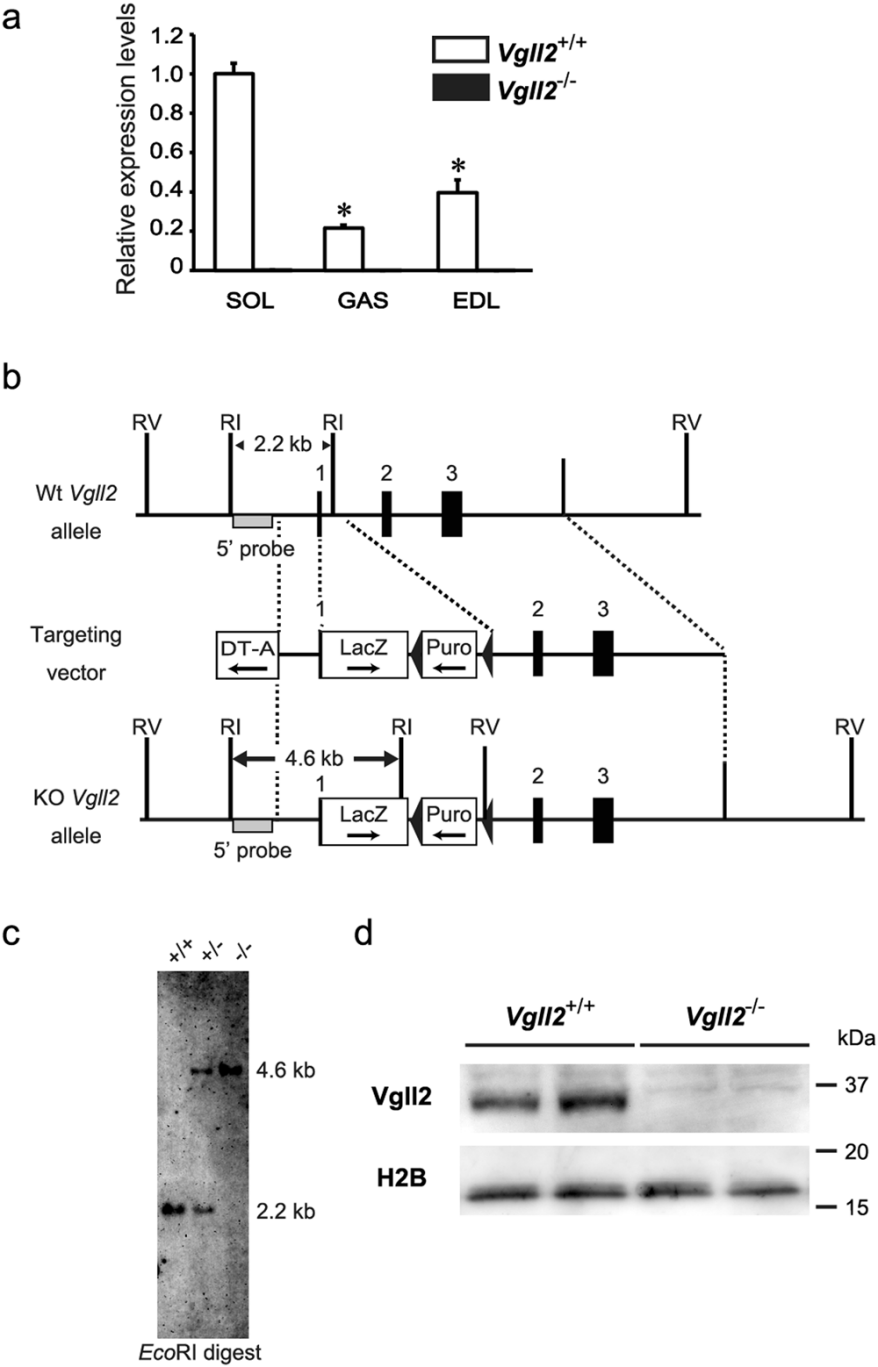
Expression patterns of *Vgll2* mRNA expression in several muscles, and generation of Vgll2-deficient mice. (**a**) *Vgll2* mRNA levels were measured by qPCR in the soleus (SOL), gastrocnemius (GAS), and extensor digitorum longus (EDL) muscles from 12-week-old *Vgll2* ^+/+^ and *Vgll2* ^−/−^ mice (n = 6). *P < 0.05 *vs. Vgll2* ^+/+^ soleus muscles. Data are presented as mean ± SEM. For comparison, the expression level in *Vgll2* ^+/+^ soleus was arbitrarily set at 1. (**b**) Schematic diagram showing the strategy used to generate the *Vgll2* null allele. The top row depicts the wild-type *Vgll2* allele, which consists of three exons (*solid boxes*). The initiation Met codon for *Vgll2* exists in exon 1. The second row depicts the targeting construct. The third row depicts the mutated *Vgll2* allele. The *lacZ* expression cassette with a SV40 polyadenylation signal, which was followed by the loxP-flanked puromycin resistant gene expression cassette (Puro) in the reverse orientation, was fused to the initiation codon of *Vgll2*. The diphtheria toxin A expression cassette (DT-A) was included at the 5′-end of the targeting vector. The external probe used for Southern blot analysis lies outside the 5′-homologous arm (*gray box*). RI, *Eco*RI; RV, *Eco*RV. (**c**) Southern blot analysis for genotyping wild-type (*Vgll2* ^+/+^), heterozygous Vgll2-deficient (*Vgll2* ^+/−^), and homozygous Vgll2-deficient (*Vgll2* ^−/−^) animals. Digestion with *Eco*RI of *Vgll2* ^+/+^ and *Vgll2* ^−/−^ alleles gave rise to fragments of 2.2 and 4.6 kb, respectively. An uncropped original image is shown in Supplementary information. (**d**) Vgll2 protein levels were assessed by Western blotting using nuclear protein extracts obtained from the soleus muscle of 12-week-old *Vgll2* ^+/+^ and *Vgll2* ^−/−^ mice. Histone H2B served as a loading control. Analyses were performed on four mice per genotype. Data from two mice per genotype are shown. Uncropped original images are shown in Supplementary information.

Previous studies suggested a role for Vgll2 in the MyHC expression during myogenic differentiation of C2C12 myoblasts^29^, but the potential function of Vgll2 in adult muscle is unknown. To elucidate the function of Vgll2 *in vivo*, we first examined whether *Vgll2* mRNA displays preferential expression patterns between individual skeletal muscles that differ in fiber type composition in 12-week-old mice. The expression level of *Vgll2* mRNA was significantly higher in the Type I and IIa fiber-enriched slow soleus muscle than in the Type IIb fiber-enriched fast gastrocnemius and extensor digitorum longus (EDL) muscles (Fig. 1a). Hence, we speculated that Vgll2 may play a role in the control of the specification of adult muscle fiber-type.

### Generation of Vgll2-Deficient Mice

To determine the influence of Vgll2 deficiency *in vivo*, we generated Vgll2 knockout mice with targeted replacement of the *Vgll2* loci by the lacZ gene in D3 ES cells (Fig. 1b). To construct a targeting vector, we obtained DNA fragments encoding *Vgll2* from a mouse EB3 cell genomic DNA library^31, 32^. Homologously recombined ES colonies and heterozygous mice were verified by Southern blot analysis (Fig. 1c). After mating heterozygous mice, all genotypes, *Vgll2* ^+/+^, *Vgll2* ^+/−^, and *Vgll2* ^−/−^ were identified (52, 106, and 45, *x* ^2^ = 0.68, chi-square test), which segregated according to the laws of Mendelian inheritance, indicating no marked embryonic lethality. Quantitative real-time PCR (qPCR) analysis revealed that *Vgll2* transcript was absent in skeletal muscle from *Vgll2* ^−/−^ mice (Fig. 1a). Moreover, Vgll2 protein was detected only in the nuclear extracts prepared from the *Vgll2* ^+/+^ soleus muscle by Western blotting with anti-Vgll2 antibody (see “Materials and Methods”), but not in *Vgll2* ^−/−^ (Fig. 1d). Compensatory responses in other Vgll family genes such as *Vgll3* and *Vgll4*, which are also expressed in skeletal muscle, were not observed in *Vgll2* ^−/−^ mice (Supplementary Fig. S1).

### Vgll2 Deficiency Induces a Slow-to-Fast Transformation of Muscle Fiber-Type

**Figure 2:**
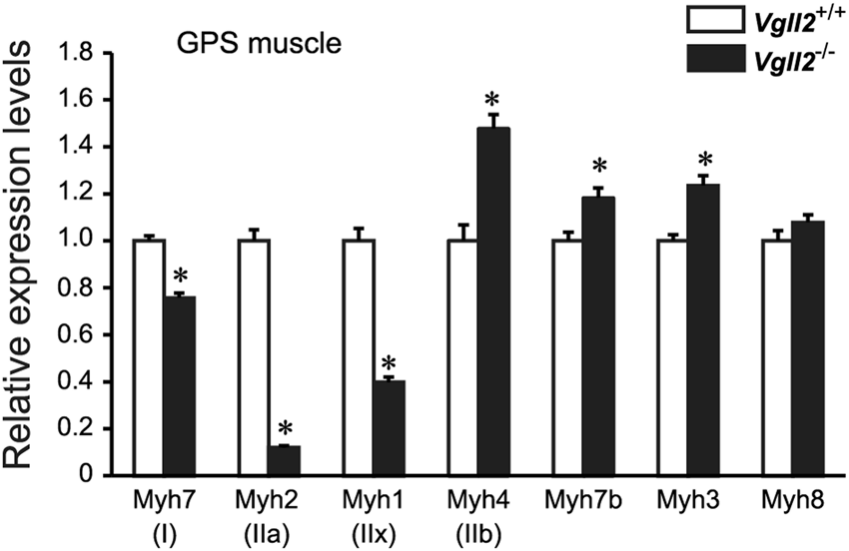
Expression analysis of myosin heavy chain isoforms in neonatal skeletal muscle. Expression levels of MyHC isoforms were measured by qPCR in the gastrocnemius-plantaris-soleus (GPS) muscle complex at postnatal day 7 (P7) of *Vgll2* ^+/+^ and *Vgll2* ^−/−^ mice (n = 8). *Myh7* (encoding MyHCI), *Myh2* (MyHCIIa), *Myh1* (MyHCIIx), *Myh4* (MyHCIIb), *Myh7b* (slow-tonic MyHC), *Myh3* (embryonic fast isoform, MyHC-emb), and *Myh8* (perinatal fast isoform, MyHC-pn) expression levels were examined. For comparison, the expression level of these genes in *Vgll2* ^+/+^ mice was arbitrarily set at 1. Data are presented as mean ± SEM. *P < 0.05 *vs. Vgll2* ^+/+^ muscles.

**Figure 3:**
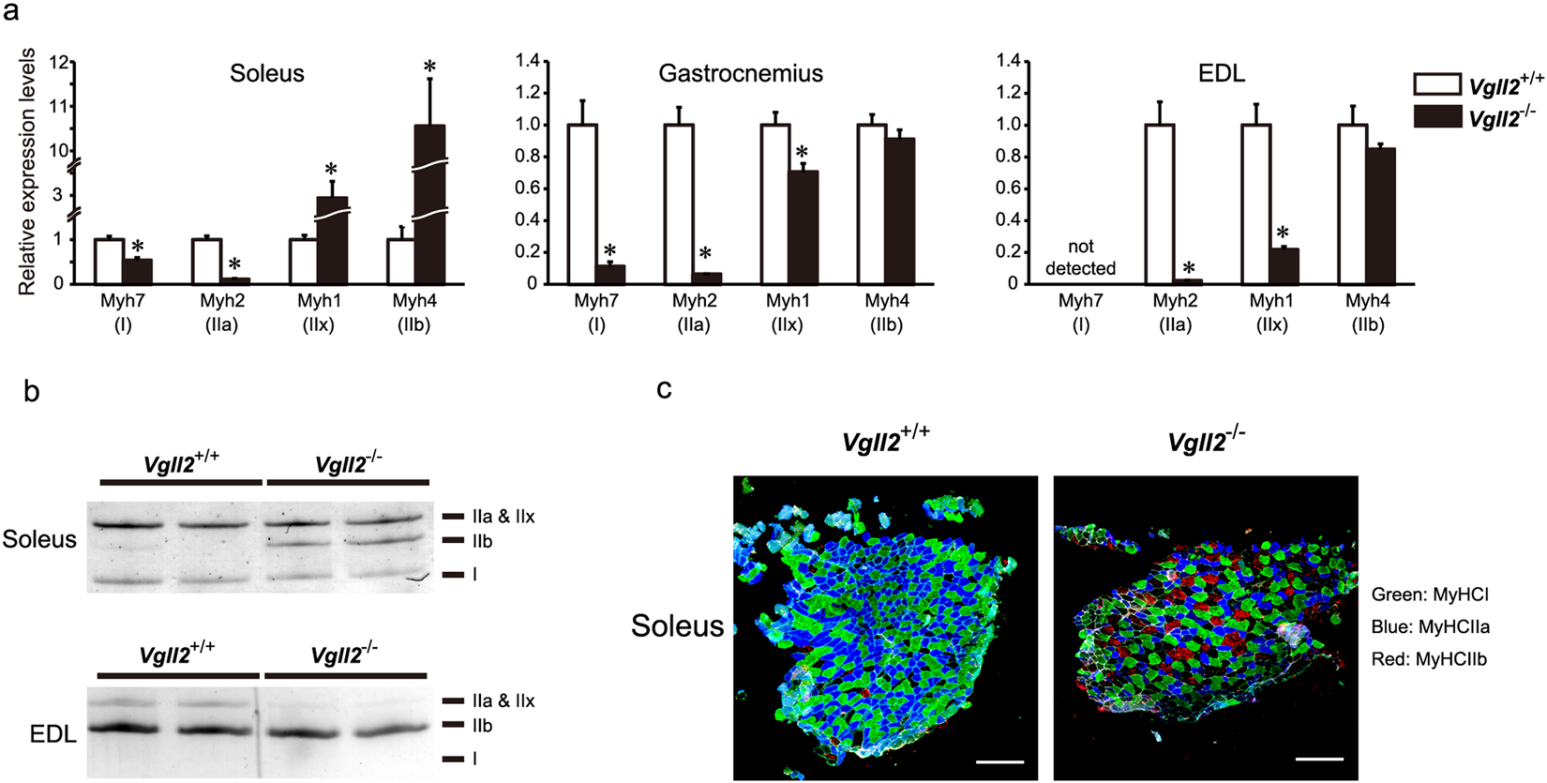
Fiber type composition analysis based on the expression of myosin heavy chain isoforms in adult skeletal muscles. (**a**) Expression levels of genes encoding MyHC isoforms, *Myh7* (I), *Myh2* (IIa), *Myh1* (IIx), and *Myh4* (IIb) were measured by qPCR in the soleus, gastrocnemius, and EDL muscles from 12-week-old *Vgll2* ^+/+^ and *Vgll2* ^−/−^ mice (n = 6). For comparison, the expression level of these genes in *Vgll2* ^+/+^ mice was arbitrarily set at 1. Data are presented as mean ± SEM. *P < 0.05 *vs. Vgll2* ^+/+^ in each muscle. (**b**) High resolution gel electrophoresis for MyHC isoform separation of protein extracts isolated from the soleus and EDL muscles of 12-week-old *Vgll2* ^+/+^ and *Vgll2* ^−/−^ mice. Equal amounts (50 ng) of total protein were separated on an 8% acrylamide gel containing glycerol. The gel was then stained with the silver stain method. Uncropped original images are shown in Supplementary information. (**c**) Representative images of immunostained soleus muscles isolated from 12-week-old *Vgll2* ^+/+^ and *Vgll2* ^−/−^ mice (n = 3). Scale bar: 200 μm.

*Vgll2* ^−/−^ mice displayed no overt abnormalities. The preferential accumulation of *Vgll2* mRNA in the slow soleus muscle led us to investigate the fiber type composition in mice lacking Vgll2. To characterize the fiber type in *Vgll2* ^−/−^ mice, we measured the relative expression levels of several MyHC isoforms by qPCR in neonatal and adult skeletal muscles. In *Vgll2* ^−/−^ gastrocnemius-plantaris-soleus (GPS) muscle at postnatal day 7 (P7), expression levels of *Myh7*, encoding slow type I myosin, were significantly reduced by 24% (Fig. 2), whereas the expression of the fast MyHC isoform gene, *Myh4*, was significantly increased (Fig. 2). These alterations in MyHC expression profiles were also observed in 12-week-old *Vgll2* ^−/−^ mice. In *Vgll2* ^−/−^ slow twitch soleus muscle, expression levels of *Myh7* were significantly decreased by 47%, whereas fast myosin genes, *Myh1* and *Myh4* were markedly increased 2.9- and 10.5-fold (Fig. 3a). We did not detect significant changes in the expression levels of all MyHC isoforms in *Vgll2* ^+/−^ soleus muscle (Supplementary Fig. S2). In addition, in the fast gastrocnemius and EDL muscle groups, expressing low levels of *Vgll2* mRNA, *Myh7*, *Myh2*, and *Myh1* expression levels were significantly reduced, whereas *Myh4* expression was sustained (Fig. 3a). Next, we determined the relative content of MyHC isoform type I, IIa (and/or IIx), and IIb by gel electrophoresis and immunostaining of each skeletal muscle (Fig. 3b,c and Supplementary Fig. S3). In *Vgll2* ^−/−^ soleus muscle, a muscle normally rich in type I and IIa fibers, both type I and type IIa fibers were reduced, and an obvious increase in type IIb fibers was detected (Fig. 3b,c and Supplementary Fig. S3). In *Vgll2* ^+/+^ soleus muscle, type IIb fibers were very rarely detected (Fig. 3b,c and Supplementary Fig. S3). Moreover, *Vgll2* ^−/−^ EDL myosin electrophoretogram showed that fast type IIa and IIx myosin were predominantly replaced by the fastest type IIb myosin (Fig. 3b), consistent with the changes in *Myh2* and *Myh1* expression levels (Fig. 3a). Thus, these observations suggest that a slow-to-fast contractile phenotype transition may occur in Vgll2-deficient mice.

### Reduced Exercise Endurance in Vgll2 KO Mice

**Figure 4:**
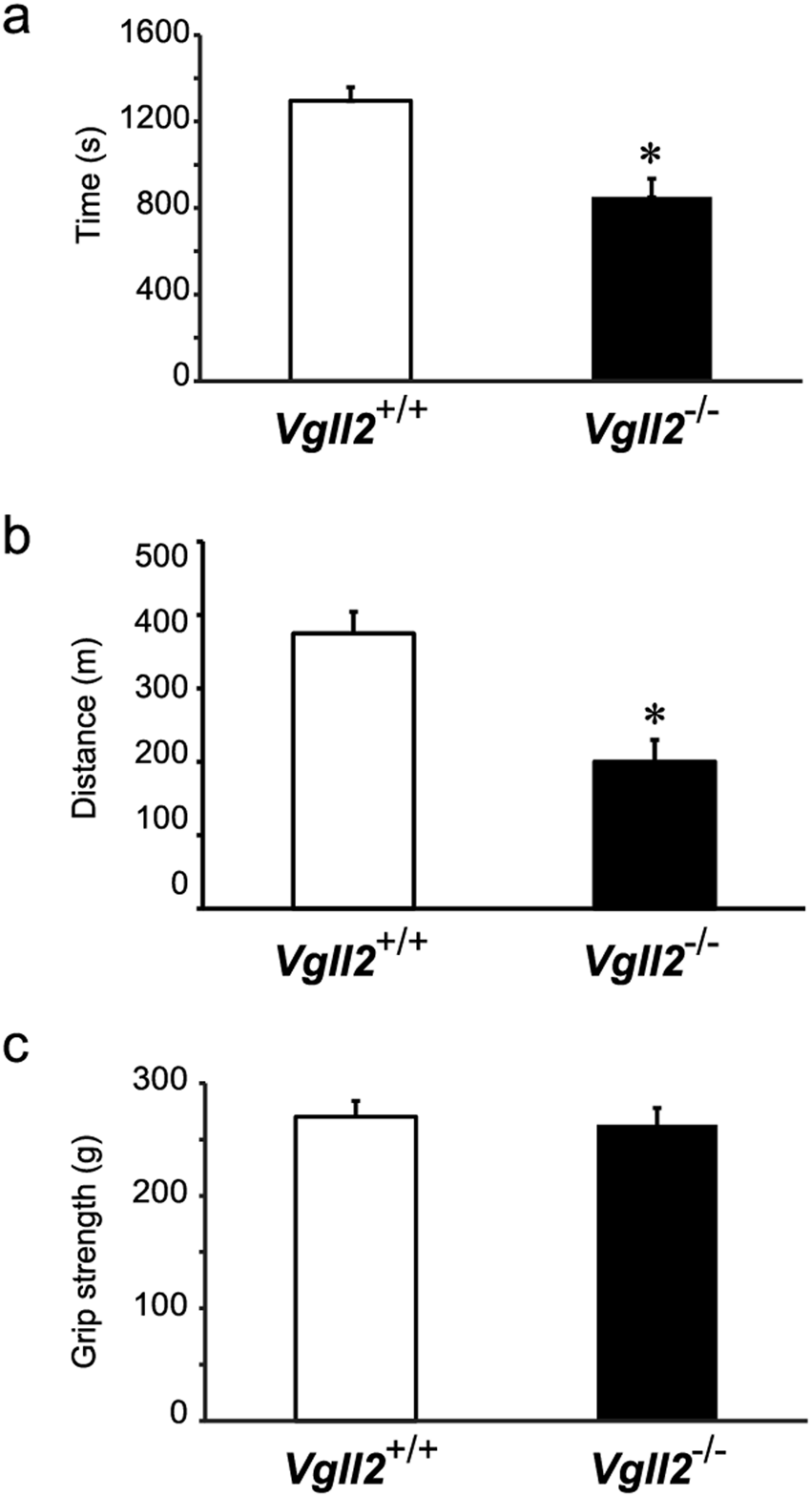
Analysis of muscle functions in Vgll2 null mice. (**a**,**b**) Physical endurance was measured by using a treadmill running test. After acclimation, mice ran on a 10% slope with a protocol using increasing speed until mice were exhausted. Exhaustion was defined as the inability of the mice to remain on the treadmill despite physical prodding. Running time (**a**) and speed were measured, and the distance (**b**) was calculated. (**c**) Skeletal muscle strength was assessed by using the grip strength test. Grip strength was measured in each mouse 20 times and the highest value of each experiment was used. Male mice (11–13-week-old) were used in all experiments (n = 8). Data are presented as mean ± SEM. *P < 0.05 *vs. Vgll2* ^+/+^ mice.

Changes in contractile properties of skeletal muscle have a considerable impact on physical activity. We therefore assessed the endurance capacity and grip strength of mice under involuntary conditions. Muscle endurance in *Vgll2* ^+/+^ and *Vgll2* ^−/−^ mice were evaluated by treadmill running to exhaustion. After acclimatization, mice were allowed to run on the treadmill set to a 10% incline at increasing speed until the mice were unable to remain on the treadmill despite prodding. *Vgll2* ^−/−^ mice were able to run for a significantly shorter time than *Vgll2* ^+/+^ mice did. *Vgll2* ^+/+^ mice ran for an average of 21.6 min, while *Vgll2* ^−/−^ mice ran for 14.2 min (Fig. 4a). Correspondingly, the running distance was significantly lower for *Vgll2* ^−/−^ mice compared to that ran by *Vgll2* ^+/+^ mice. The distance covered by *Vgll2* ^−/−^ mice was 200.3 meters on average, whereas *Vgll2* ^+/+^ mice ran 374.7 meters on average (Fig. 4b). Grip strength was not reduced in *Vgll2* ^−/−^ mice (Fig. 4c). These results indicate that, in skeletal muscles, Vgll2 contributes greatly to exercise endurance.

### Direct Binding of Vgll2 to TEF/TEAD in the Skeletal Muscle

**Figure 5:**
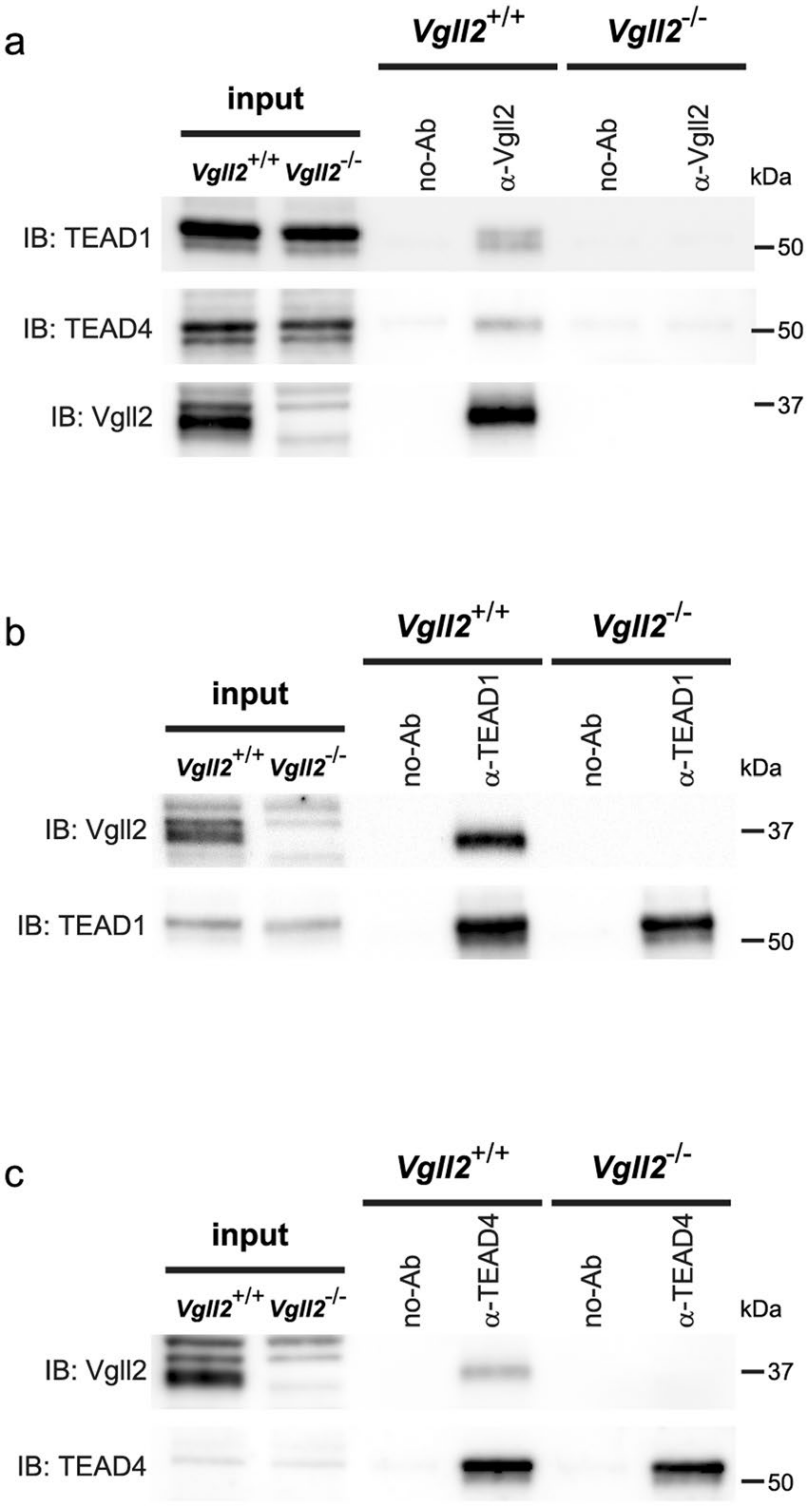
Physical interaction analysis between endogenous Vgll2 and TEAD1 in skeletal muscles at the neonatal stage. (**a**) Immunoprecipitation was conducted with the anti-Vgll2 antibody followed by Western blot analysis to detect TEAD1, TEAD4 and Vgll2 in GPS muscle extracts from *Vgll2* ^+/+^ and *Vgll2* ^−/−^ mice at P7. (**b**) Immunoprecipitation was conducted with an anti-TEAD1 antibody followed by Western blot analysis to detect Vgll2 and TEAD1 in P7 GPS muscle extracts. (**c**) Immunoprecipitation was conducted with an anti-TEAD4 antibody followed by Western blot analysis to detect Vgll2 and TEAD4 in P7 GPS muscle extracts. Control samples were GPS muscle lysates incubated without anti-Vgll2 or -TEAD1 antibody (no-Ab). Uncropped original images are shown in Supplementary information.

Vgll2 possesses an evolutionarily conserved TDU motif, which binds to TEAD transcription factors. Previous studies suggested a role for TEF/TEAD family member, TEAD1 and TEAD4 in the regulation of slow muscle gene expression^30, 33–36^. Vgll2 deficient mice showed fiber type composition changes in skeletal muscle and reduced endurance. Our findings suggest that Vgll2 may be required for TEAD-mediated muscle fiber-type regulation, as one of the cofactors. To find the physical interaction between Vgll2 and TEAD1 in skeletal muscles, we conducted co-immunoprecipitation experiments using extracts from neonatal (P7) GPS muscles. As expected, endogenous TEAD1 and TEAD4 proteins were efficiently co-immunoprecipitated by the anti-Vgll2 antibody only in *Vgll2* ^+/+^ extracts, but not in Vgll2 KO extracts (Fig. 5a). In addition, reciprocal co-immunoprecipitation with an anti-TEAD1 antibody precipitated endogenous Vgll2 only in *Vgll2* ^+/+^ extracts (Fig. 5b). Similarly, co-immunoprecipitation with an anti-TEAD4 antibody precipitated endogenous Vgll2 only in *Vgll2* ^+/+^ extracts (Fig. 5c). Together, our findings raise the possibility that Vgll2 plays a cooperative role in TEAD1/4-associated transcription in skeletal muscle through protein binding.

To examine whether Vgll2 deficiency alters the expression levels of other TEAD cofactors such as Yap and Taz^27, 37, 38^, we quantified these genes by qPCR. However, we did not detect significant changes in the expression levels of these genes (Supplementary Fig. S4).

### Downregulation of miR-208b Expression in Vgll2 Deficient Skeletal Muscle and Increase in its Target Proteins

**Figure 6:**
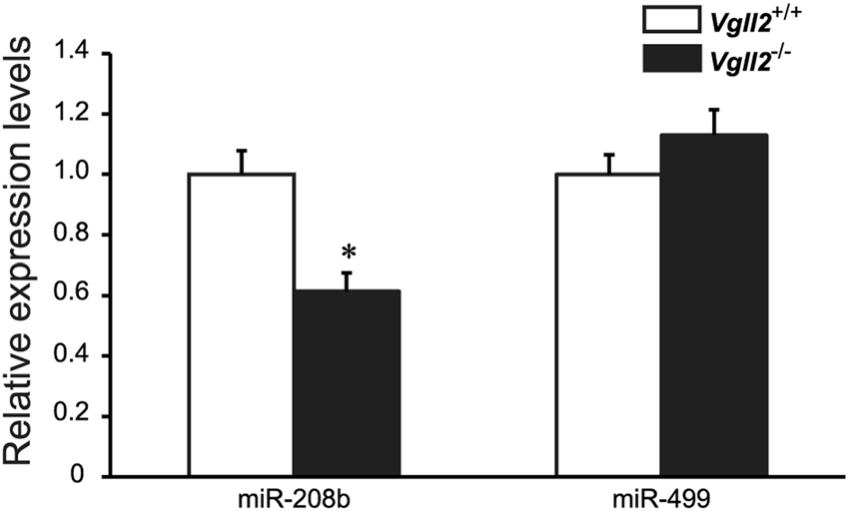
Expression analysis of miRNAs in the neonatal skeletal muscle. miR-208b and miR-499 expression levels in the GPS muscle from *Vgll2* ^+/+^ and *Vgll2* ^−/−^ mice at P7 (n = 8). For comparison, the expression level of these miRNAs in *Vgll2* ^+/+^ mice was arbitrarily set at 1. Data are presented as mean ± SEM. *P < 0.05 *vs*. *Vgll2* ^+/+^ muscles.

We next sought to investigate the molecular mechanisms underlying slow-to-fast fiber-type transition in *Vgll2* ^−/−^ mice. Previous studies showed that two miRNAs, miR-208b and miR-499, serve as fiber-type modulators by inhibiting the activity of transcriptional repressors of genes encoding slow-twitch contractile proteins such as Sox6, Sp3, and Purβ^21, 39–42^. Although these miRNAs share a comparable seed sequence and target the same mRNAs, they are differentially regulated: miR-208b is encoded within the *Myh7* gene, and miR-499 is encoded within *Myh7b* gene^43^. We measured the relative expression levels of these miRNAs in neonatal skeletal muscles by qPCR. At P7, consistent with gene expression results of their host MyHC mRNAs (Fig. 2), the expression level of miR-208b was significantly reduced in *Vgll2* ^−/−^ GPS muscles by 39%, whereas miR-499 expression was slightly increased (Fig. 6).

**Figure 7:**
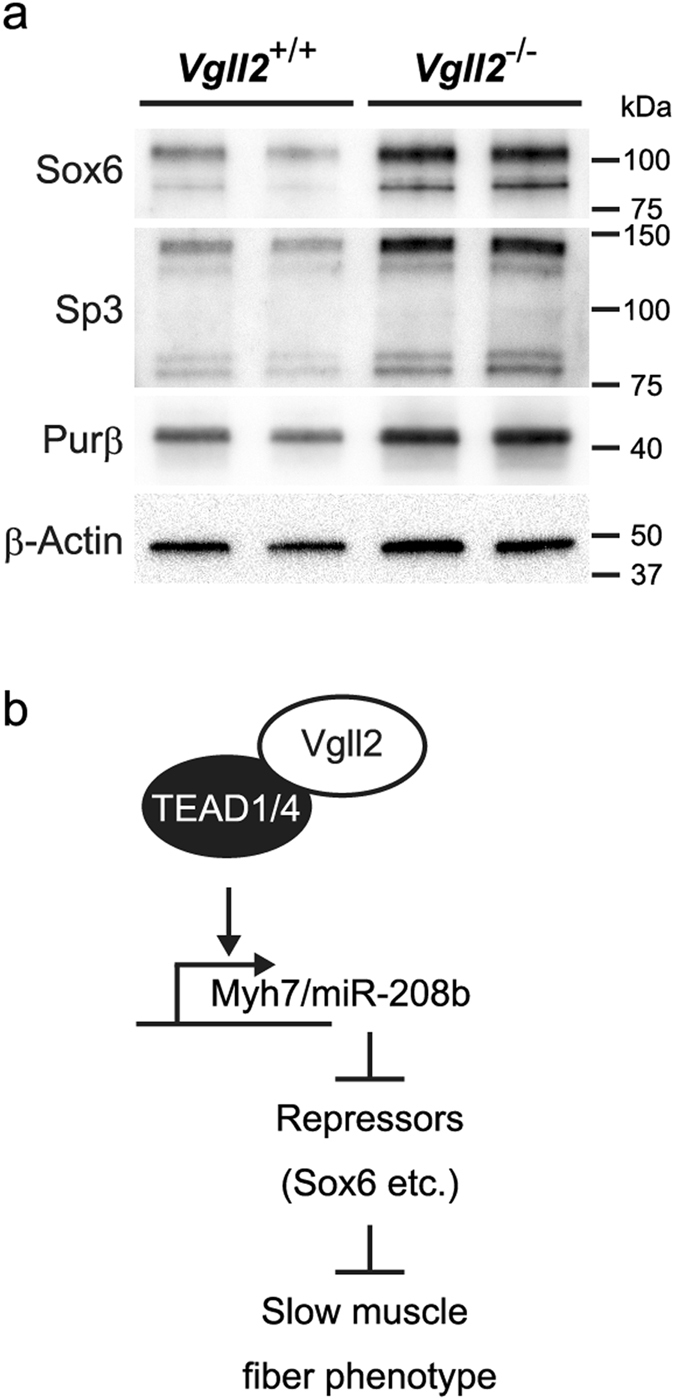
Expression analysis of transcriptional repressor proteins in the neonatal skeletal muscles. (**a**) Sox6, Sp3, and Purβ protein levels were assessed by Western blotting in the GPS muscle from *Vgll2* ^+/+^ and *Vgll2* ^−/−^ mice at P7. β-Actin was used as a loading control. Analyses were performed on four mice per genotype. Data from two mice per genotype are shown. Uncropped original images are shown in Supplementary information. (**b**) Schematic representation of the possible mechanism of TEAD1-mediated Vgll2 functions in muscle fiber type regulation.

In order to further validate the reduction in miR-208b in *Vgll2* ^−/−^ skeletal muscles, we compared protein levels of its downstream targets, Sox6, Sp3, and Purβ by Western blotting analysis. In *Vgll2* ^−/−^ GPS muscles at P7, Sox6 and Sp3 protein levels were markedly increased, while Purβ protein level was moderately increased (Fig. 7a). Collectively, our findings suggest that Vgll2 directly and indirectly participates in the specification of mature skeletal muscle fiber characteristics.

## Discussion

In the present study, we provide the first *in vivo* evidence that the transcription cofactor, Vgll2, plays a role in muscle fiber-type distribution and physical performance. The Absence of Vgll2 results in shifts in fiber type of skeletal muscle toward a faster phenotype and decreases in slow isoform MyHC gene and its intronic miRNA, *Myh7/miR-208b*, and deregulation of miR-208b targets. As physical interaction between Vgll2 and TEAD1/4 were detected in neonatal muscles, we propose a possible mechanism involved in the programing of slow muscle fibers through the formations of Vgll2-TEAD1/4 complex (Fig. 7b).

Although several *in vitro* studies provided evidence that Vgll2 promotes skeletal muscle differentiation in cultured muscle cells^26, 28^, its *in vivo* role in this capacity has not been investigated in skeletal muscle tissues. Herein, we show that Vgll2 is involved in the establishment and maintenance of adult skeletal muscle phenotypes. At P7, before the establishment of adult muscle fiber phenotype, gene expression analysis by qPCR revealed that Vgll2 deficient skeletal muscle presented a faster MyHC profile. Furthermore, this faster MyHC profile was maintained in adult mice. Both transcript and protein levels of MyHCIIa in adult *Vgll2* ^−/−^ mice were dramatically decreased in slow and fast twitch muscles when compared to that in WT mice. Similarly, reduced expression of MyHCI was observed at both the transcript and protein levels. The changes in fast isoforms of MyHC expression were prominent in the slow soleus muscle. Transcript and protein levels of MyHCIIx and IIb were dramatically increased. On the other hand, MyHCIIx expression was significantly decreased while that of MyHCIIb was sustained in fast muscle groups such as the gastrocnemius and EDL. Taken together, our experimental results show that Vgll2 may be required not only for the establishment, but also for the maintenance of the adult slow muscle phenotype. A conditional knockout approach may serve to clear Vgll2 function in skeletal muscle development and exclude the possibility of the loss of Vgll2 during development that might affect muscle fiber type distribution in adult mice.

Muscle fiber composition is an important determinant of muscle endurance in animals, along with the capacity for mitochondrial fuel oxidation and ATP synthesis^44–46^. Consequently, slow-to-fast fiber-type transformation often leads to a reduction in exercise endurance^47–49^. In the treadmill running experiments, the running distance and time were significantly decreased in *Vgll2* ^−/−^ mice, indicating that genetic deletion of Vgll2 impaired the endurance capacity of the mice. On the other hand, there was no difference in grip strength between *Vgll2* ^−/−^ and control animals, suggesting that Vgll2 activity is dispensable for the maintenance of muscle integrity. Thus, slow-to-fast fiber type transformation of skeletal muscle in *Vgll2* ^−/−^ mice has distinct effects on physical activity.

Previous reports showed that Vgll2 can activate TEAD family transcription factors by physical interaction in *in vitro* assays^26^. We verified that Vgll2 forms a complex with TEAD1/4 in neonatal muscle. TEAD1/4 regulates the expression of muscle specific genes. Overexpression of TEAD1 induces a transition toward a slow muscle contractile phenotype and satellite cell hyperplasia^30, 50^. These previous data indicate that TEAD1 are sufficient factors in both muscle specific gene expression and regeneration. However, they require additional factors or coactivators for gene expression^51^. It is noteworthy that TEAD1/4 expression is not restricted to specific tissues. Hence, skeletal muscle-specific functions are conferred by muscle-specific cofactors. In recent studies, several cofactors for TEAD family members have been identified, including YAP/TAZ, p160 family of nuclear receptor coactivators, and other Vgll members^26, 27, 37, 38, 52–54^. Vgll2 is exclusively expressed in skeletal muscles in adult animals^25, 26^. In addition, we now show that Vgll2 is more highly expressed in slow muscle groups. Thus, our data suggest the possibility that Vgll2 contribute to TEAD-dependent regulation of muscle-specific genes, especially in slow-twitch muscle.

Many striated muscle-specific genes contain muscle-specific cytidine-adenosine-thymidine (MCAT) elements in the control region^55–57^. MyHCI is also a well-established MCAT-element containing gene. Several previous studies described that the *Myh7* promoter was activated by TEAD proteins through the direct binding of TEAD proteins on MCAT sequences^58–60^. Together with our results, these data suggest that MyHCI might be one of the direct targets of the Vgll2-TEAD1/4 complex. Interestingly, miR-208b, which works as a slow type regulator in a miRNA-mediated transcriptional regulatory network, was also decreased in the GPS muscles of *Vgll2* ^−/−^ neonates in parallel with *Myh7* expression. miR-208b represses negative regulators of slow-twitch muscle genes such as *Sox6*, *Sp3*, and *Pur*β^21^. In our study, the expression of Sox6, Sp3, and Purβ proteins was increased in Vgll2 deficient GPS muscles. As miR-208b uses the *Myh7* promoter^21^, the Vgll2-TEAD1 complex may also regulate miR-208b expression through *Myh7* promoter regulation.

In our gene expression analysis, MyHCIIa was remarkably decreased in Vgll2-deficient muscles. Impairment of exercise tolerance in Vgll2-deficient mice could be induced by downregulation of not only MyHCI, but also MyHCIIa because type IIa fibers are mitochondria rich and are resistant to fatigue. Thus, MyHCIIa might be a target of Vgll2. Although several studies described the regulatory mechanisms of MyHCIIa expression^8, 61, 62^, MCAT and its related elements have not been identified in the MyHCIIa regulatory region. Additional studies are required to understand how Vgll2-associated transcription pathway participates in this regulation. The expression of *Myh1*, encoding MyHCIIx, is also significantly decreased in Vgll2 deficient GPS muscles at P7. Given that *Myh1* is one of the Sox6 target genes^41^, it raises the possibility that the decrease in *Myh1* expression is associated with an increase in Sox6 protein. Thus, both direct and indirect involvements of Vgll2 are proposed for muscle fibers regulation.

This study sheds light on the role for Vgll2 in slow-twitch myofiber gene expression *in vivo*. After determination by basal regulation, muscle fiber type is modified in response to external stimuli like muscle usage. Previous studies implicated TEAD1 in the exercise-induced fiber type remodeling^30^. Additional experiments are necessary to elucidate the participation of Vgll2 in the regulation of exercise-induced fast-to-slow fiber type transition. Furthermore, previous studies pointed out the possibility that Vgll2 can interact with another transcription factor, MEF2C^26^. Further analyses are warranted to fully investigate Vgll2 interaction factors and its downstream target genes.

In summary, this study provides the first *in vivo* evidence that Vgll2 participates in muscle fiber-type specification and in the maintenance of normal muscle fiber composition at the adult stage under basal conditions. Moreover, our findings suggested the direct and indirect involvement of the Vgll2-TEAD1 complex in the regulatory program for slow skeletal muscle gene expression.

## Methods

### Antibody Production

Peptides corresponding to residues 293 to 308 (QSLGLSVDSGKRRREC) of mouse Vgll2 were synthesized as an immunogen for the production of polyclonal antibodies in rabbits using a customized service (MBL, Nagoya, Japan). After verification of Vgll2 specificity by ELISA and Western blotting, the antiserum was affinity-purified against the peptide.

### Construction of the Targeting Vector

The targeting construct (Fig. 1b) contained 1.3 kb and 7.4 kb of *Vgll2* homologous sequences on the 5′ and 3′ regions of the *lacZ* expression cassette with a SV40 polyadenylation signal, which followed the Vgll2 initiation codon. The loxP-flanked puromycin-resistance gene expression cassette (Puro) driven by a phosphoglycerate kinase promoter was inserted in the reverse orientation after the *lacZ* expression cassette. The diphtheria toxin A expression cassette (DT-A) was included at the 5′-end of the targeting vector for negative selection.

### Generation of Vgll2-Deficient Mice

The D3 mouse embryonic stem cells^63^ were electroporated with the targeting vector and selected in medium containing puromycin. Targeted clones were identified by Southern blotting using the DIG High prime DNA Labeling and Detection Starter Kit II (Roche, Basel, Switzerland) with 5′-external probes. Chimeric mice were obtained by injection of positive ES cells into blastocysts and crossed with wild-type C57BL/6 J mice (Japan SLC, Hamamatsu, Japan) to test for germline transmission of the disrupted *Vgll2* allele. The genotypes of offspring were examined by Southern blotting analysis. To establish a mouse line carrying the disrupted *Vgll2* allele, heterozygous mice were backcrossed with C57BL/6 J mice. The mouse experiments were approved by the Animal Care and Use Committee of the National Cerebral and Cardiovascular Center in Japan, and were performed in accordance with the institutional and national guidelines and regulations.

### RNA Isolation, Reverse Transcription, and Quantitative Real Time PCR

Total RNA was extracted with miRNeasy mini Kit (QIAGEN, Hilden, Germany). For mRNA analysis, cDNA was synthesized with SuperScript III Reverse Transcriptase (Invitrogen, Carlsbad, CA, USA) using 2 μg of total RNA and random hexamers (Invitrogen). For miRNA analysis, cDNA was synthesized with Universal cDNA Synthesis Kit II (Exiqon, Vedbaek, Denmark) using 10 ng of total RNA. cDNA was amplified, using either Power SYBR Green PCR Mater Mix (Applied Biosystems, Foster City, CA, USA), Eagle Taq Master Mix with ROX (Roche), or ExiLENT SYBR Green master mix (Exiqon), with a 7900HT Fast Real Time PCR System (Applied Biosystems). The primer sequences used in this study were as follows: *Vgll2*: forward (F) 5′-CAGCAGCAAAGCACACAGAAG-3′ and reverse (R) 5′-TACGCGCTGTTCCAGAAGG-3′; *Myh7*: (F) 5′-CTACAGGCCTGGGCTTACCT-3′ and (R) 5′-TCTCCTTCTCAGACTTCCGC-3′; *Myh2*: (F) 5′-ATCCAAGTTCCGCAAGATCC-3′ and (R) 5′-TTCGGTCATTCCACAGCATC-3′; *Myh1*: (F) 5′-ATGAACAGAAGCGCAACGTG-3′ and (R) 5′-AGGCCTTGACCTTTGATTGC-3′; *Myh4*: (F) 5′-AGACAGAGAGGAGCAGGAGAGTG-3′ and (R) 5′-CTGGTGTTCTGGGTGTGGAG-3′; *Myh7b*: (F) 5′-AGAGTGTGGAGCAGGTGGTATT-3′ and (R) 5′-GGTCTGATTGATTCGAGAAACC-3′; *Myh3*: (F) 5′-TGAACAGATTGCCGAGAACG-3′ and (R) 5′-GGAGAATCTTGGCTTCTTCGTG-3′; *Myh8*: (F) 5′-ATCGTGAGAACCAGTCCATCC-3′ and (R) 5′-TTTGCCAGACTCCTCCTTCTTC-3′, *Vgll3*: (F) 5′-CAGGGAGACATTGGGTCAGT-3′ and (R) 5′-TGGTCCAAAAGGAAGTTGGA-3′, *Vgll4*: (F) 5′-CTACCGGAGACCACCCAGT-3′ and (R) 5′-GCAAAGTGGTCATCCACTGA-3′,Yap: (F) 5′-CCCGACTCCTTCTTCAAGC-3′ and (R) 5′-CTCGAACATGCTGTGGAGTC-3′, and Taz: (F) 5′-GAAGGTGATGAATCAGCCTCTG-3′ and (R) 5′-GTTCTGAGTCGGGTGGTTCTG-3′. Taqman Gene Expression Assay for mouse *ACTB* (beta actin) endogenous control (4352341E) was purchased from Applied Biosystems. For miRNA analysis, the following predesigned primer sets were purchased from Exiqon and used: *U6* (203907); hsa-miR-208b-3p (204636); and hsa-miR-499a-5p (205935). *ACTB* and *U6* were used as reference genes for normalization of mRNA and miRNA level, respectively.

### Western Blotting Analysis

For total protein extracts, muscles isolated from both legs of each mouse were homogenized in buffer (50 mM Tris-HCl, pH 7.6, 150 mM NaCl, 1 mM EDTA, 1% NP-40, 0.5% sodium deoxycholate, 0.1% SDS) supplemented with protease inhibitor (Complete Ultra Mini Protease inhibitor cocktail tablets used as instructed; Roche) and phosphatase inhibitor (PhosSTOP phosphatase inhibitor cocktail tablets used as instructed; Roche) using a BioMasher II homogenizer (Nippi, Tokyo, Japan). ProteoExtract Subcellular Proteome Extraction Kits (Merck Millipore, Darmstadt, Germany) were used according to the manufacturer’s instructions with some modifications to prepare nuclear extracts. Briefly, snap frozen muscles isolated from both legs of each mouse were homogenized directly in extraction buffer II containing Protease Inhibitor cocktail. After centrifugation, the cytoplasmic fraction (supernatant) was transferred to a new tube, and the pellet was resuspended in extraction buffer III containing protease inhibitor cocktail and benzonase endonuclease. After centrifugation, the nuclear fraction (supernatant) was collected. Protein concentrations were determined using BCA Protein Assay Kit (Thermo Scientific, Rockland, IL, USA) or DC Protein Assay Kit (Bio-Rad, Hercules, CA, USA). After adding 5× SDS sample buffer (300 mM Tris-HCI, pH 6.8, 10% SDS, 25% β-mercaptoethanol, 0.05% bromophenol blue, 50% glycerol), samples were boiled for 5 min, and subjected to SDS-PAGE. Proteins in the gels were transferred to a PVDF membrane using a Trans-Blot Turbo Blotting System (Bio-Rad). Following a blocking step with PVDF Blocking Reagent for Can Get Signal (TOYOBO, Osaka, Japan), the membrane was incubated with primary antibody and probed with the appropriate HRP-conjugated secondary antibodies. The membranes were developed using ECL Prime Western Blotting Detection Reagent (GE Healthcare, Little Chalfont, UK), and chemiluminescent signals were detected by a LAS-1000 image analyzer (FUJIFILM, Tokyo, Japan). The antibodies used in this study were as follows: anti-Vgll2 (described above, 1:1000), Histone H2B (Merck Millipore, 07–371, 1:1000), Sox6 (Abcam, Cambridge, UK, ab30455, 1:1000), Sp3 (Santa Cruz Biotechnology, Dallas, TX, USA, sc-644, 1:1000), Purβ (Bethyl Laboratories, Montgomery, TX, USA, A303-650A, 1:1000), β-Actin (Cell Signaling Technology, Danvers, MA, USA, #4970, 1:1000), and anti-rabbit IgG (horseradish peroxidase-linked) and anti-mouse IgG (horseradish peroxidase-linked) (Cell Signaling Technology).

### High Resolution Gel Electrophoresis

Protein extraction and high resolution gel electrophoresis for MyHC isoform separation were performed as described previously^64^. Briefly, muscle tissues were isolated from both legs per mouse was homogenized in buffer (0.1 M Tris-HCl, pH 8.0, 10% SDS, 40 mM DTT, 5 mM EDTA) supplemented with protease inhibitor cocktail (Complete Ultra Mini Protease inhibitor cocktail tablets used as instructed; Roche) using a BioMasher II homogenizer (Nippi), and 50 ng of protein sample was separated on 8% acrylamide gel containing glycerol by 140 V for 22 h at 4 °C. The gel was then stained with Silver Stain II Kit (Wako) according to the manufacturer’s instructions.

### Immunofluorescence

Immunohistochemistry techniques were used for fiber type determination. Soleus muscles were harvested and frozen in liquid nitrogen cooled isopentane. Frozen sections (7 μm) were cut in a cryostat on microscope slides. Slides were allowed to warm up to room temperature, fixed in 4% paraformaldehyde-PBS for 20 min at 4 °C, and permeabilized with 0.1% Triton X-100-PBS for 10 min at 4 °C, and immersed in 0.01 M citrate buffer (pH 3.0) at 37 °C for 30 min for antigen retrieval. Slides were then blocked with 10% normal goat serum (Nichirei, Tokyo, Japan) for 1 h at room temperature, and incubated at 4 °C for overnight with the following primary antibody cocktail: MyHCI antibody (BA-F8), MyHCIIa antibody (SC-71), and MyHCIIb antibody (BF-F3) were mixed in equal volume. Slides were then incubated for 1 h at room temperature with the following secondary antibody cocktail: Alexa Fluor 488 goat anti-mouse IgG2b (Invitrogen, A-21141, 1:200), Alexa Fluor 647 goat anti-mouse IgG1 (Invitrogen, A-21240, 1:200), and Alexa Fluor 568 anti-mouse IgM (Invitrogen, A-21043, 1:200). Images were captured under the FLUOVIEW FV10i (Olympus, Tokyo, Japan), then merged and pseudocolored in FV10-ASW (Olympus). All primary antibodies against MyHC isoforms were purchased from DSMZ (Braunschweig, Germany). Collected cell culture supernatants were used as primary antibody solutions.

### Co-Immunoprecipitation

Physical interaction between Vgll2 and TEAD1 was analyzed by co-immunoprecipi-tation analysis using Dynabeads coupled with anti-rabbit IgG or –mouse IgG according to the manufacturer’s instructions (Invitrogen). Prior to Co-IP, we incubated Dynabeads M-280 Sheep anti-Rabbit IgG or Dynabeads M-280 Sheep anti-Mouse IgG (Invitrogen) with anti-Vgll2, anti-TEAD1 (BD Biosciences, San Jose, CA, USA, 610923), or anti-TEAD4 (abcam, ab58310) antibody (5 μg per 50 μl of beads slurry), respectively, with rotation at 4 °C. For Co-IP experiments, muscle tissue was homogenized in buffer (20 mM HEPES-NaOH, pH 8.0, 150 mM NaCl, 1 mM EDTA, 1.5 mM MgCl_2_, 0.5% Triton X-100, 10% glycerol) supplemented with Complete Ultra Mini Protease inhibitor cocktail and PhosSTOP phosphatase inhibitor cocktail using a BioMasher II homogenizer. Sonication for 5 cycles of 30 sec ON/30 sec OFF with the Bioruptor UCD-300 (Tosho Electric Ltd., Yokohama, Japan) at maximal power setting of homogenates was followed by adding Benzonase Nuclease (Merck Millipore) and incubation for 1 h with rotation at 4 °C. Following centrifugation, the supernatant was rotated at 4 °C for 1 h with antibody-coupled Dynabeads, washed 3 times in PBS (pH7.4), and eluted with 50 μL sample buffer (60 mM Tris-HCl, pH 6.8, 2% SDS, 10% glycerol, 5% β-mercaptoethanol, 0.01% bromophenol blue) and denatured at 95 °C for 5 min. After denaturation, samples were subjected to SDS-PAGE. The negative control was performed using lysates with beads (no-Ab) without any antibodies. Transferring and blocking were performed as described above. The membranes were then incubated with the primary antibody against Vgll2 (1:1000), anti-TEAD1 (1:1000) and anti-TEAD4 (1:1000) overnight at 4 °C diluted in Can Get Signal solution, and probed with the appropriate HRP-conjugated secondary antibody. The secondary antibodies used in this study were as follows: anti-rabbit IgG, Conformation Specific (Cell Signaling Technology, #3678, 1:10000) and anti-Mouse IgG, Light Chain Specific (Jackson ImmunoResearch, West Grove, PA, USA,115-035-174, 1:20000).

### Treadmill Exercise

The muscle endurance test was performed as described previously^65^. In brief, we used a MK-680S treadmill (Muromachi Kikai Co., Ltd., Tokyo, Japan). For 3 days, animals were acclimated to treadmill running for 5 min at a speed of 10 m/min on a 0% grade. After acclimation, animals ran on a treadmill with a 10% uphill grade starting at a speed of 10 m/min for 5 min. Every subsequent 2 min, the speed was increased by 2 m/min until the mice were exhausted. Exhaustion was defined as the inability of the animal to remain on the treadmill despite mechanical prodding. Running time and speed were measured, and the distance was calculated.

### Grip Strength

Grip strength was measured as described previously^65^. In brief, we use a MK-380M grip strength meter (Muromachi Kikai Co., Ltd.). The grip strength of each individual mouse was measured 10 times, the same measurements were repeated on the next day, and the highest value of each experiment was used.

### Statistical analyses

Quantitative analyses were performed on at least 3 independent biological samples. Data are expressed as the mean ± SEM. Data were analyzed by using the Student’s t-test or one-way ANOVA with Dunnett post hoc test. *P* < 0.05 was considered as significant.

## Electronic supplementary material

supplementary information
