# Supplementary material for: Vestigial-like 2 contributes to normal muscle fiber type distribution in mice
Source: Sci Rep. 2017 Aug 2;7:7168. doi: 10.1038/s41598-017-07149-0 (PMC5540913; doi:10.1038/s41598-017-07149-0)
Supplement: Supplementary file 1 — supplementary information [file 41598_2017_7149_MOESM1_ESM.pdf]

Supplementary information for:

“Vestigial-like 2 contributes to normal muscle fiber type distribution in mice”

By: Masahiko Honda, Kyoko Hidaka, So-ichiro Fukada, Ryo Sugawa, Manabu Shirai, Masahito Ikawa, and Takayuki Morisaki

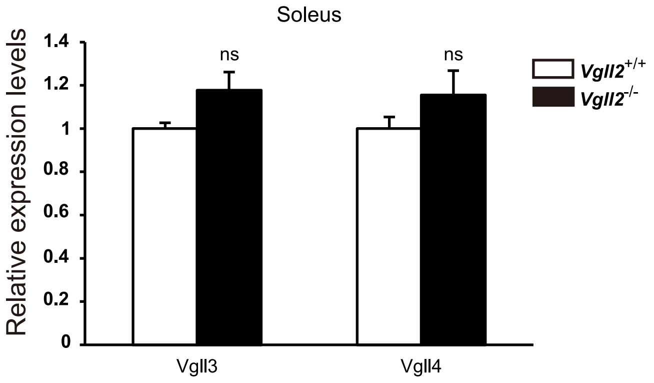

Supplementary Figure S1. Quantification of the expression levels of Vgll family genes in adult skeletal muscle. *Vgll3* and *Vgll4* mRNA levels were measured by qPCR in the soleus muscles from 12-week-old *Vgll2*$^{+/+}$ and *Vgll2*$^{-/-}$ mice (n = 6). ns, not significant difference.Data are presented as mean ± SEM. For comparison, the expression level in *Vgll2*$^{+/+}$ was arbitrarily set at 1.

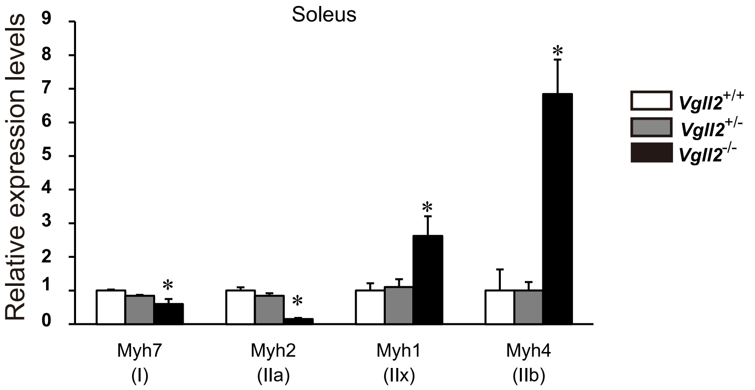

Supplementary Figure S2. Quantification of the expression levels of myosin heavy chain isoforms in adult skeletal muscle. Expression levels of genes encoding MyHC isoforms, *Myh7* (I), *Myh2* (IIa), *Myh1* (IIb), and *Myh4* (IIb) were measured by qPCR in the soleus muscles from 12-week-old *Vgll2*$^{+/+}$, *Vgll2*$^{+/-}$, and *Vgll2*$^{-/-}$ mice (n = 3). For comparison, the expression level of these genes in *Vgll2*$^{+/+}$ mice was arbitrarily set at 1. Data are presented as mean ± SEM. **P* < 0.05 vs. *Vgll2*$^{+/+}$ in each muscle.

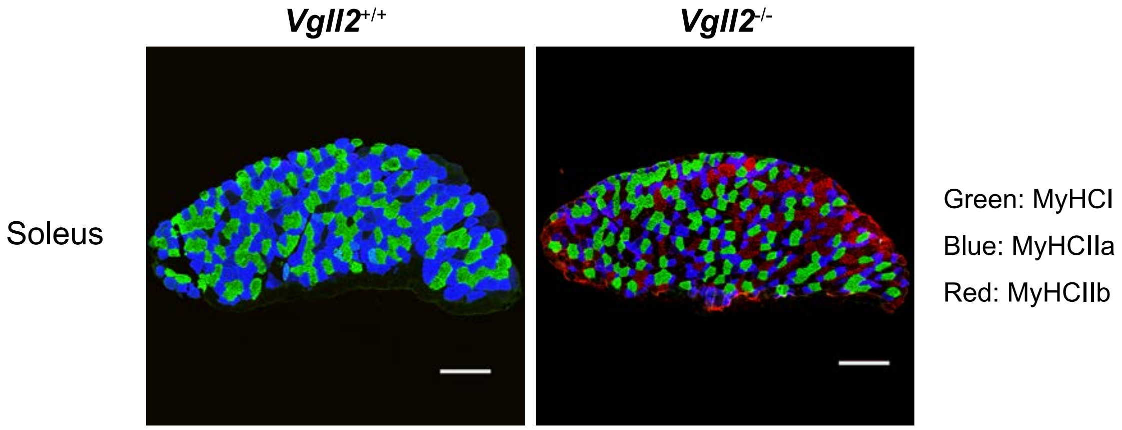

Supplementary Figure S3. Fiber type composition analysis based on the immunostaining of myosin heavy chain isoforms in adult skeletal muscles. Alternative images of immunostained soleus muscles presented in Figure 3. Samples are isolated from 12-week-old *Vgll2*$^{+/+}$ and *Vgll2*$^{-/-}$ mice. Scale bar: 200 μm.

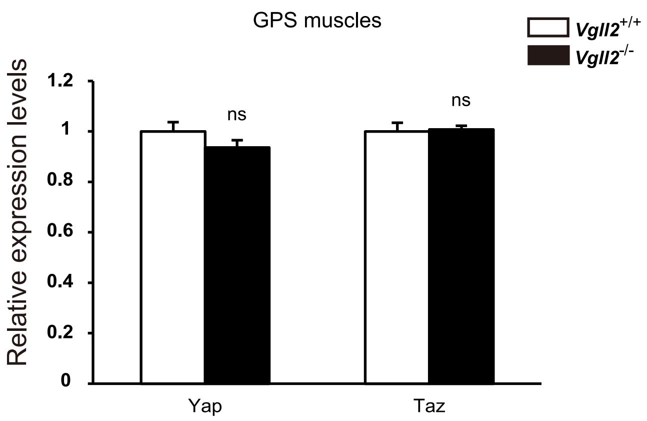

Supplementary Figure S4. Quantification of the expression levels of cofactor of TEAD transcriprtion factor genes in neonatal skeletal muscle. Expression levels of cofactors of TEAD transcription factors were measured by qPCR in the gastrocnemius-plantaris-soleus (GPS) muscle complex at postnatal day 7 (P7) of *Vgll2*$^{+/+}$ and *Vgll2*$^{-/-}$ mice (n = 8). Yap and Taz mRNA expression levels were examined. For comparison, the expression level of these genes in *Vgll2*$^{+/+}$ mice was arbitrarily set at 1. Data are presented as mean ± SEM. ns, not significant difference.

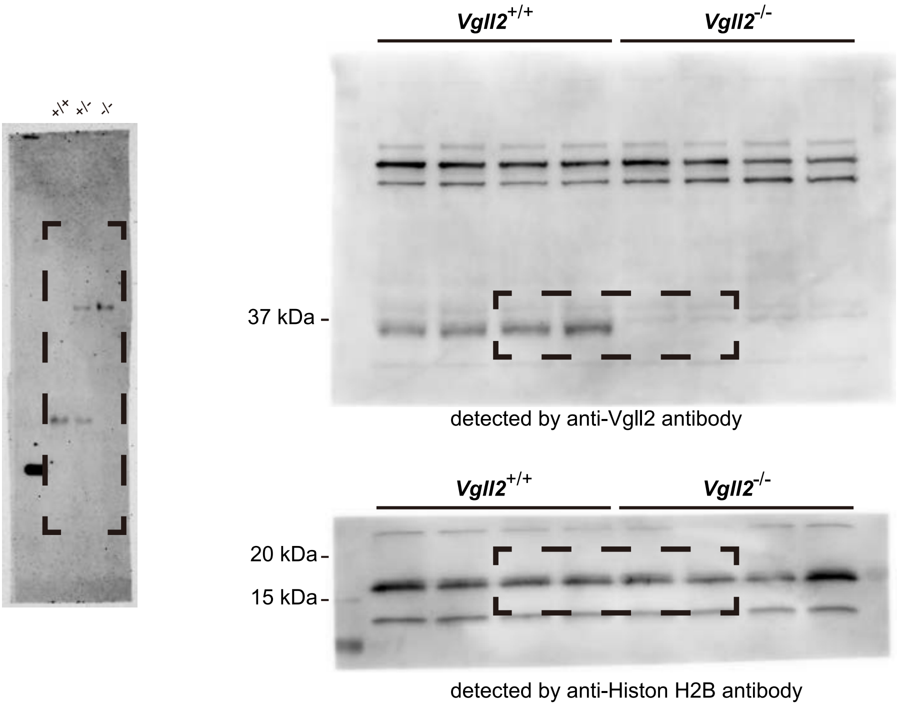

Supplementary Figure S5. Full-length blot images of Fig. 1c and d (with minimal contrast adjustment). Dotted areas were cropped, processed in accordance with the guidelines, and used for the figure.

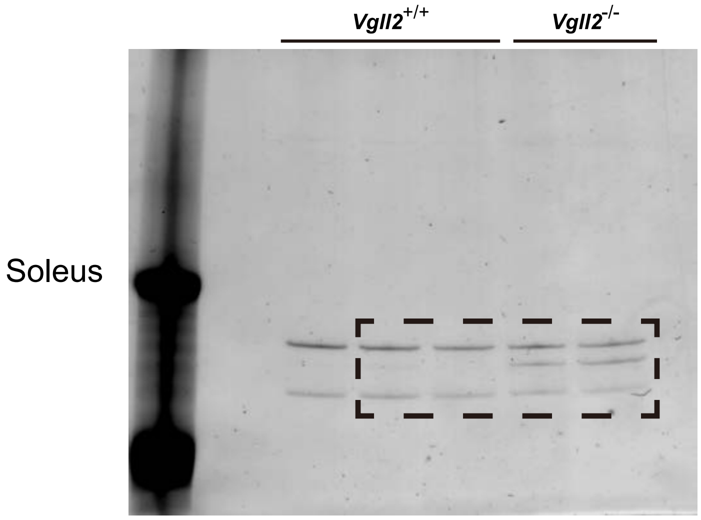

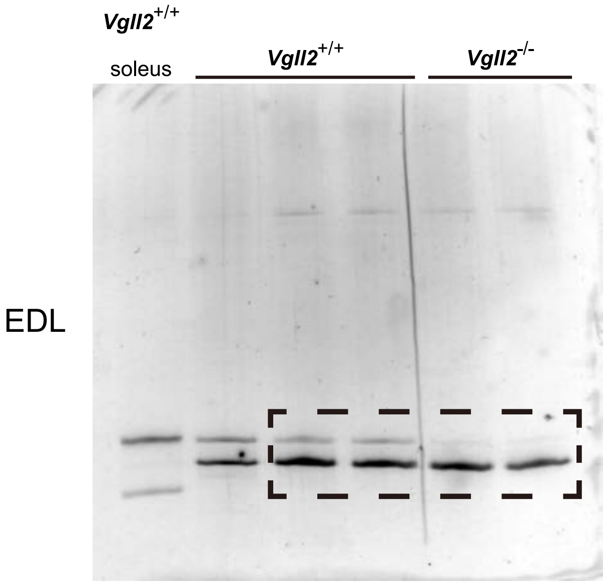

Supplementary Figure S6. Full-length gel images of Fig. 3b (with minimal contrast adjustment).

Dotted areas were cropped, processed in accordance with the guidelines, and used for the figure.

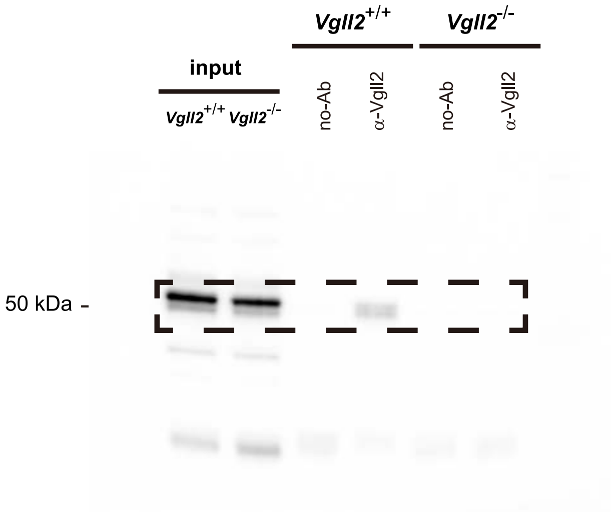

detected by anti-TEAD1 antibody

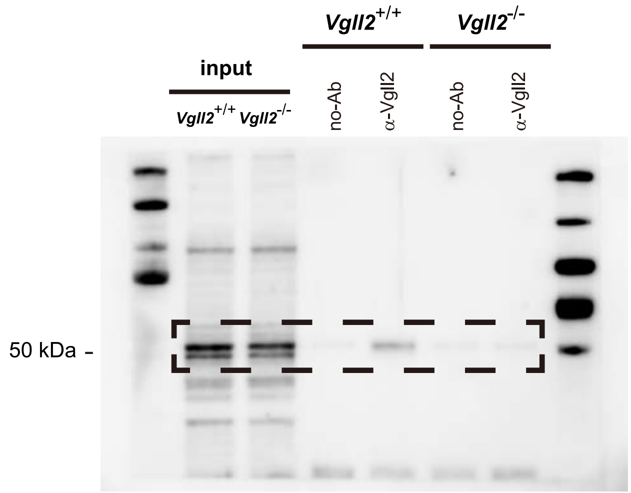

detected by anti-TEAD4 antibody

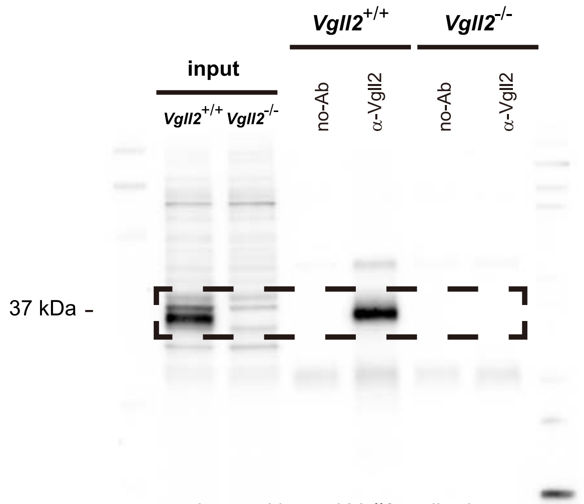

detected by anti-Vgll2 antibody

Supplementary Figure S7. Full-length blot images of Fig. 5a (with minimal contrast adjustment).

Dotted areas were cropped, processed in accordance with the guidelines, and used for the figure.

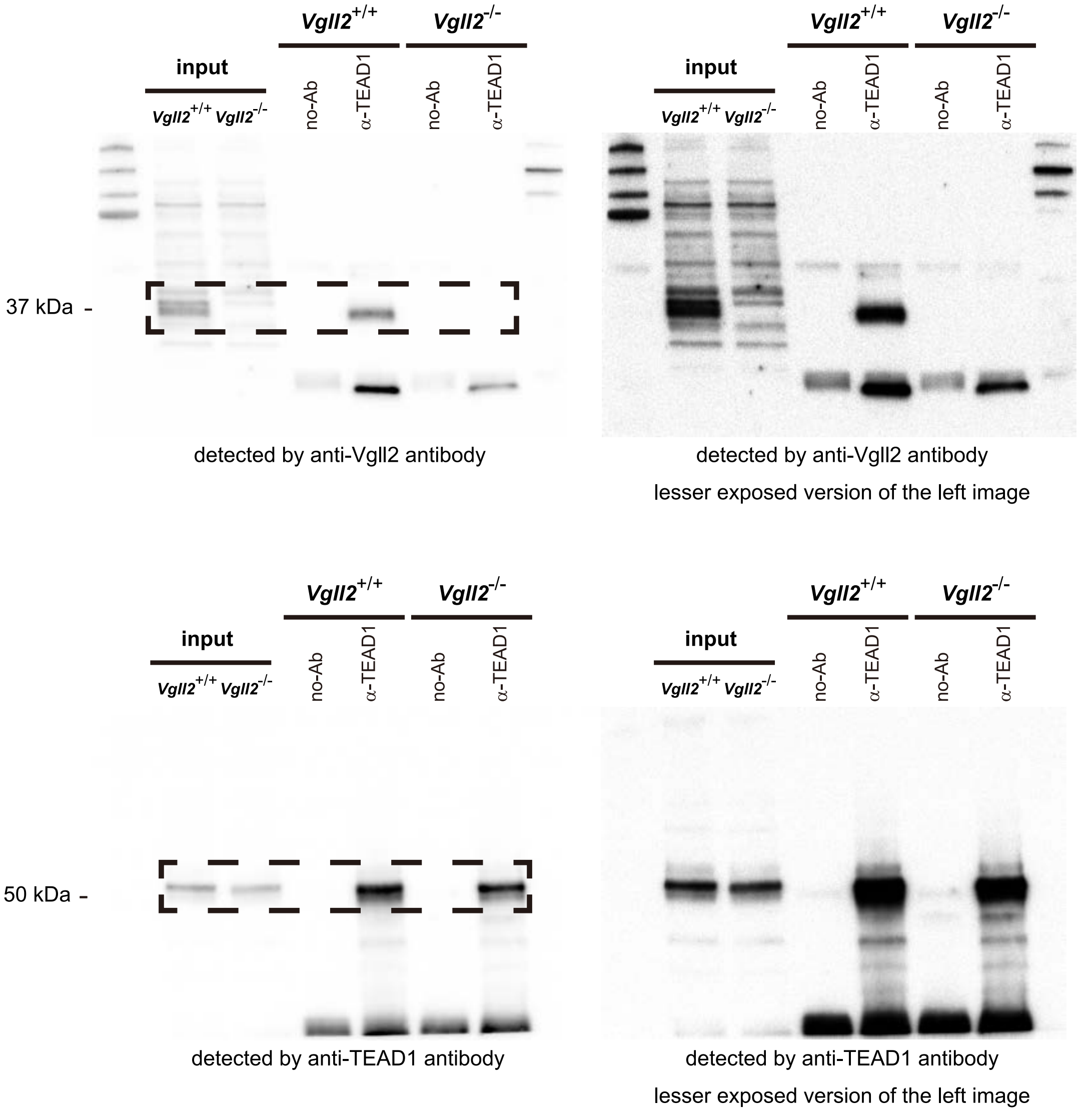

Supplementary Figure S8. Full-length blot images of Fig. 5b (with minimal contast adjustment).
Dotted areas were cropped, processed in accordance with the guidelines, and used for the figure.

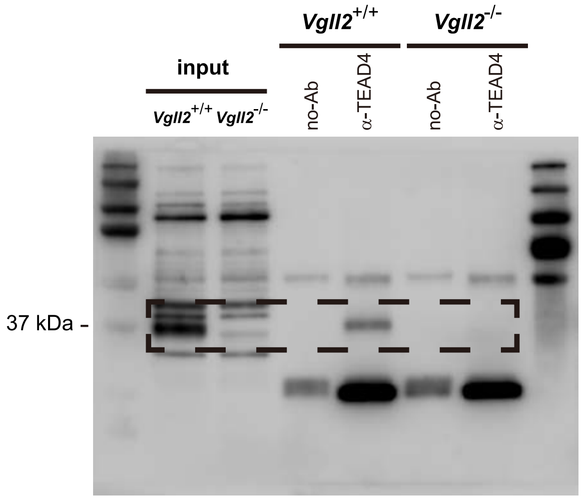

detected by anti-Vgll2 antibody

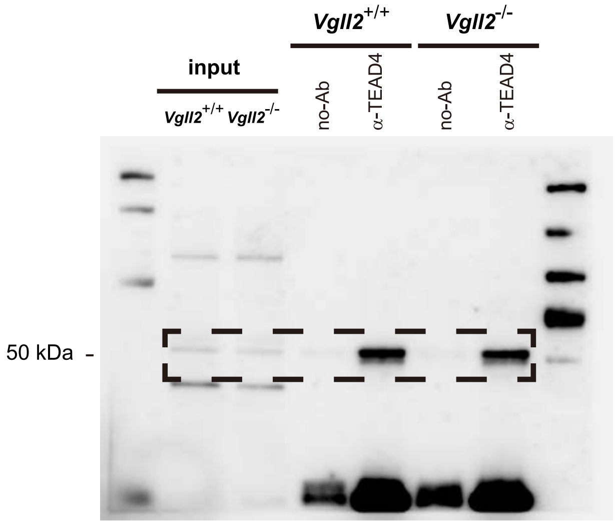

detected by anti-TEAD4 antibody

Supplementary Figure S9. Full-length blot images of Fig. 5c (with minimal contrast adjustment).

Dotted areas were cropped, processed in accordance with the guidelines, and used for the figure.

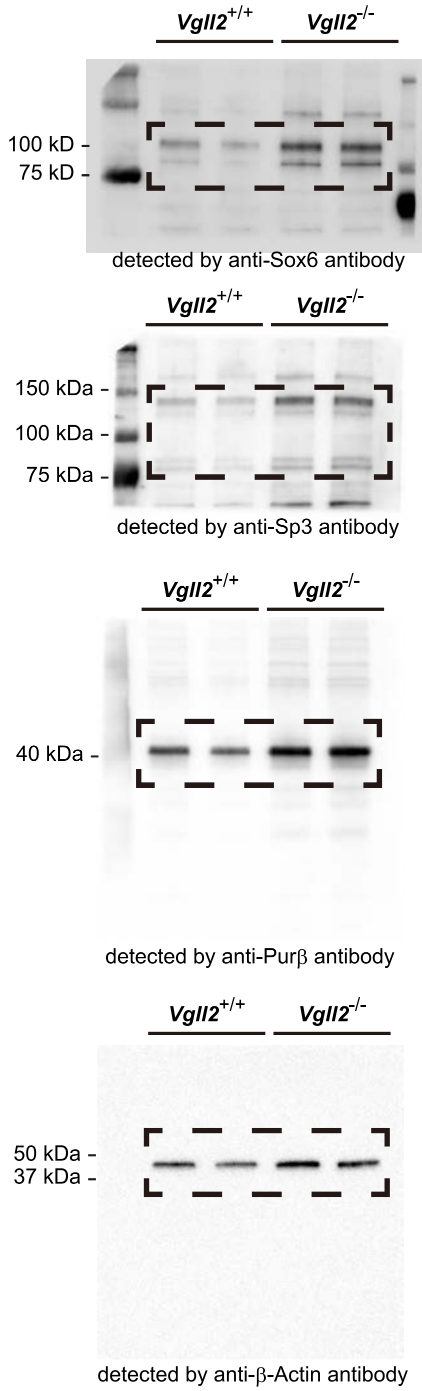

Supplementary Figure S10. Full-length blot images of Fig. 7a (with minimal contrast adjustment).
Dotted areas were cropped, processed in accordance with the guidelines, and used for the figure.
